# A real-time communication and information system for triage, positioning, and documentation (TriPoD) in mass-casualty incidents: a qualitative observational study

**DOI:** 10.1186/s12873-025-01274-0

**Published:** 2025-07-06

**Authors:** Veronica Lindström, Klara Jepsen, Sara Heldring, Torkel Kanfjäll, Monica Rådestad

**Affiliations:** 1 Department of Nursing, Division of Ambulance Service Faculty of Medicine, Umeå University, Region Västerbotten, Umeå, 901 87 Sweden; 2 Division of Nursing, Department of Neurobiology, Care Sciences and Society, Karolinska Institutet, Huddinge, Sweden; 3 Samariten Ambulans AB, Stockholm, Sweden; 4 Department of Health Promoting Science, Sophiahemmet University, Stockholm, Sweden; 5 Department of Clinical Science and Education, Karolinska Institutet, Södersjukhuset, Stockholm, Sweden; 6 Capio S:t Görans hospital, Stockholm, Sweden

**Keywords:** Decision support techniques, Command and control, Communication, Mass-causal incidents, Situational awareness, Triage

## Abstract

**Background:**

In mass-casualty incidents (MCIs), command centers often rely on oral or written reports, leading to communication gaps, misunderstandings, and inadequate logistics of available resources. This study developed a real-time communication and information system for Triage, Position, and Documentation (TriPoD) via action research in collaboration with end-users to ensure high usability. TriPoD integrates commercially available technology, utilizing a digital triage tag with a unique ID that attaches to each individual with an injury. Emergency medical service (EMS) providers scan the electronic triage tag (e-triage tag) via a mobile app, instantly sending data to command centers through a web portal. The developed TriPoD enables seamless sharing of patient information from the MCI scene during transport to and within hospitals.

**Aim:**

This study aimed to explore the usability of TriPoD during a simulated MCI with figurants.

**Methods:**

A qualitative observational design was employed, with non-participant observers stationed at the incident site, a regional command center, and a hospital command center. The observers compared TriPoD with standard procedures and management, and collected user perspectives on the system evaluated. A thematic analysis was used to guide the analysis.

**Results:**

The results revealed that command centers receive real-time updates on patient count, triage status, and location faster and with more accurate numbers of injuries than traditional methods do. Data transmitted through the web portal was updated each time a new patient was scanned, enabling continuous real-time monitoring and informed decision-making. EMS providers and command center users observed TriPoD usability, with delays when EMS providers did not consistently scan injured individuals.

**Conclusion:**

This study demonstrates that seamless information sharing from the scene of an MCI enhances reliable communication and management efforts. Although TriPoD shows strong potential for improving MCI response and management, further development, testing, and collaboration with intended end-users are essential for its continued improvement. The study was approved by the Swedish Ethical Review Authority (No: 2023-04615-01). International Registered Report Identifier (IRRID): PRR1-10.2196/57819. Clinical trial number: Not applicable.

**Supplementary Information:**

The online version contains supplementary material available at 10.1186/s12873-025-01274-0.

## Introduction

In light of uncertainties, including conflicts and mass-casualty incidents (MCIs), the need for preparedness in response to MCIs and disasters is critical. Efforts have been made to measure and evaluate the efficiency of emergency response, incident management, and decision-making in the setting of MICs and disasters [1–3]. The ability to cope with a sudden high load of casualties, regardless of the type of incident, presents multiple challenges. An essential incident management task is coordinating patient flow and allocating resources to ensure efficient patient care [1]. Communication is another core task of Incident Commanders (IC) [1]. Effective communication and information sharing are crucial first steps in improving situational awareness at all management levels, and a lack of coordination can be a significant obstacle. This has been highlighted in several systematic reviews, reports, and inquiries [2, 4, 5]. Therefore, it is essential to identify potential information gaps during MCI simulations and training [6]. Inaccurate information can lead to inaccurate decision-making, which may decrease the probability of survival for the injured in an MCI. Decision-making is essential for handling multiple challenges, and in the MCI context, these decisions are time-sensitive. Different models have described how ICs process information and act under pressure. The information processing model views decision-making as an iterative and dynamic cognitive process [7]. Dynamic decision-making encompasses situations where individuals must make a series of interconnected decisions within an environment that changes over time [8], for example, in the context of an MCI. Events with dynamic decision-making are often complex due to time delay between actions, decisions, and outcomes, as well as decisions interacting with and influencing each other in unpredictable ways [8]. In contrast, the recognition-primed decision-making model emphasizes how experienced ICs make rapid judgments based on pattern recognition [9]. Additionally, dual-process theory distinguishes between fast, intuitive responses and slower, analytical reasoning in decision-making [10]. A recent study showed insight into how ICs adapted their strategies and decision-making under extreme conditions by shifting from instantaneous to automatic decisions [3]. Therefore, there is still a need for real-time information systems that ensure the seamless transfer of critical information, such as triage status, casualty counts, and the location of the injured, to ICs, thereby minimizing information gaps that could delay or misguide life-saving decisions. Nevertheless, disaster medical leaders and responders must possess multifaceted competencies [11]. There is a need for a more robust competency framework, which has been emphasized as essential for supporting effective performance, particularly within disaster medical leadership [11]. Five challenges have been identified in managing MCIs or disasters. One challenge is the presence of unconfirmed, incomplete, or contradictory information, such as the number of injuries [2]. These challenges can significantly impact decision-making during incident commands, which are often exacerbated by a lack of real-time information. Effective and timely information sharing within the chain of command is crucial for effective command and control, as well as optimal resource utilization [12]. Limited information is available because Emergency Medicine Service (EMS) providers primarily use radio or phones to communicate and share information from the scene. Despite many efforts to support prehospital communication from the scene of an incident to command centers, inefficiencies persist [13, 14]. Correct, effective, and timely prehospital communication and information sharing regarding the number of injuries, triage categories, injured patients’ conditions, position, and transport facilitate an adequate medical response and enable receiving hospitals to make appropriate preparations, thereby avoiding bottlenecks in their systems [12, 15]. Providing real-time information is a field where digital technology can excel in MCI and disaster management [16]. The development of digital techniques to provide real-time information sharing to incident commands in prehospital and hospital settings is imperative to effectively respond to MCIs and disasters [12, 13, 17]. The Incident Command System (ICS) and its structure (e.g., chain of command) vary across countries [1, 2]. However, no ICS is inherently better than others [1], and the benefits of command structures with an “all-hazards approach” are highlighted in the literature [18, 19]. Despite the lessons learned and technological advancements, we must continue to explore what constitutes effective management, information sharing, and communication [1, 11, 13].

**Fig. 1:**
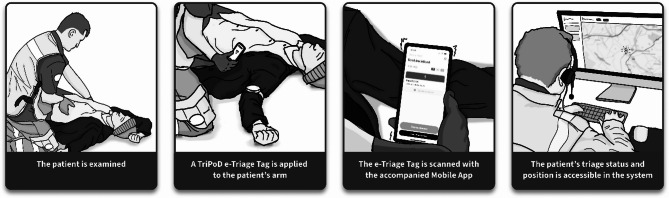
Emergency medical services (EMS) providers’ workflow using TriPoD e-triage tag, picture by Oscar Bergström, Nordforce Technology AB

**Fig. 2:**
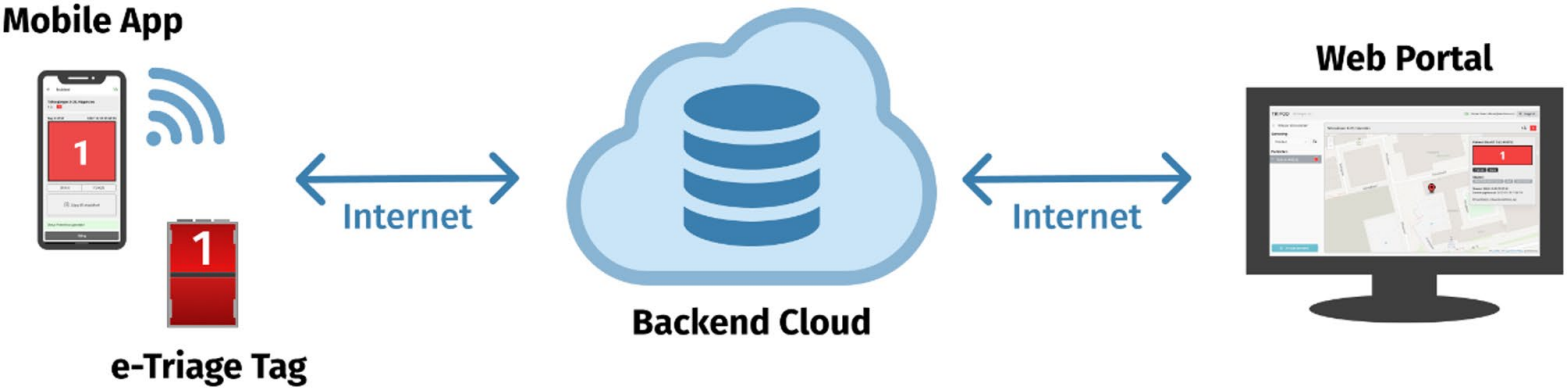
Overview of information transmission in TriPoD, picture by Oscar Bergström Nordforce Technology AB

Triage during MCIs is supported by various tools, ranging from traditional paper-based systems to increasingly advanced electronic triage (e-triage) solutions. While early e-triage systems have relied on RFID tags, barcodes, or mobile technology, recent developments encompass a broader spectrum of digital tools. These include scoring-based systems for pandemic response [20], Internet of Things (IoT)-based vital sign monitoring and e-triage [21], and telemedicine-supported triage enhanced by machine learning [22]. These innovations aim to improve prioritization, situational awareness, and communication across care levels. Despite rapid technological advances, challenges persist. Many vendors offer electronic systems to facilitate real-time information sharing and support decision-making. However, the effectiveness of such systems depends on their ability to meet the needs of users working under time pressure, uncertainty, and limited resources. Although mobile applications are increasingly used in EMS, a recent review found limited research on mobile triage solutions for MCI and/or disaster contexts [17]. Moreover, the need for improved organization and prehospital triage systems has been identified as a research priority [23]. Regardless, digital systems must be simple, intuitive, and usable in the field while ensuring that critical patient information can be transferred seamlessly throughout the entire care chain, from the incident site to hospital admission, supporting continuity, coordination, and clinical relevance. Therefore, in this study, a real-time communication and information system for Triage, Position, and Documentation (TriPoD) was developed. Development has taken place with the support of action research (AR) in collaboration with end-users to ensure high usability [24]. The definition of usability refers to how easy and pleasant a system is for users to carry out specific tasks within a defined environmental context [25]. The TriPoD system is a digital solution developed specifically for the project, utilizing an e-triage tag with a unique ID attached to each patient. While some components incorporate standardized technologies, including open-source software, the overall system is not based on any overarching open-source platform. The system includes a web-based portal that is accessible through standard web browsers, including Chrome, Firefox, Safari, and Edge, on desktops, laptops, or tablets. The portal provides a real-time situational overview, including incident locations and triaged patient data. Triage data is initially stored securely on the mobile device and then uploaded to the TriPoD Cloud System, which is specific to the operating country or region. Once successfully transferred, the data is purged from the mobile device. Access to the cloud-stored data via the mobile application or web portal requires user authentication through a system account with appropriate credentials and permissions. As the system remains in a prototype phase, it has not yet implemented healthcare-specific data standards such as HL7 or FHIR. The process of using TriPoD (as showed in Figs. 1 and 2) starts with EMS providers scanning the e-triage tag via a mobile app, instantly sending data, and visualizing the information for command centers through a web portal. In the event of an internet outage, data is stored locally within the mobile application and automatically uploaded to the web portal once connectivity is re-established. The triage colours selected on the e-triage tag remain visible for inspection without requiring access to the mobile application and can be changed manually if the need for re-triage arises.

TriPoD was developed to enable real-time information sharing from the MCI scene during transport to and within hospitals. At hospitals, seamless data transitions occur between the e-triage tag applied at the scene and a tracking tag adapted for hospital environments. This data transition is necessary due to the lack of a national standard for data transfer between different healthcare providers, and the transition is crucial for ensuring information sharing throughout the entire healthcare chain. In this way, on-scene management and hospital management can work together in a seamless process. This seamless integration between digital systems may also support informed decision-making and enhance situational awareness at various levels of management.

This study focuses on the user’s perception of using the TriPoD system in simulated settings, including the MCI, at the scene of the incident, at a regional command center, and a hospital command center. Although similar projects may be in progress, to our knowledge, no system was fully implemented at the time of this study. This study aimed to explore the usability of TriPoD during a simulated MCI with figurants.

## Methods

### Study design and study background

A qualitative observational design was employed to explore the usability of TriPoD during a simulated MCI with figurants in 2024. A thematic analysis was conducted, and the Standard for Reporting Qualitative Research (SRQR) guided the presentation of the study [26]. The study has been approved by the Swedish Ethical Review Authority (No: 2023-04615-01), and an action research study protocol has been registered in the International Registered Report Identifier (IRRID): PRR1-10.2196/57819. Clinical trial number: Not applicable. Through action research, researchers have ensured collaboration and developmental work with end-users, and these end-users have actively contributed to shaping the developed solutions, ensuring that the outcomes are meaningful and actionable in their specific contexts [27]. During pilot tests and exercises, attitudes, motivations, regulations, and organizational challenges related to interoperability and information sharing at all levels of MCI management have been investigated [24].

### Setting

In the event of an MCI, Sweden employs a national management model that encompasses management levels and roles, as well as standardized terminology. The management model comprises the Regional Command Center (RCC), the Hospital Incident Command Group (HICG), and the EMS command unit at the scene of the MCI. The duty officers at the RCC oversee regional coordination and resource allocation during an MCI. The Swedish National Board of Health and Welfare regulates EMS, which assigns the county councils the responsibility for the ambulance service. The EMS operates as a pool of regional resources, encompassing a variety of agencies, healthcare providers, personnel, equipment, and facilities, all under the direction of the regional authority. The study was performed within the Stockholm County EMS organization and involved one hospital participating in the MCI scenario. The usability of TriPoD was tested in a realistic field exercise, including a scenario with an antagonistic event. The exercise included EMS command units, ambulances (staffed with two EMS providers), hospital staff from the emergency department (ED), X-ray department, operating and intensive care units, surgery departments, the HICG, and the RCC. All EMS providers were equipped with mobile devices and e-triage tags. They received one hour of instruction about the TriPoD system and using the e-triage tag before the exercise began. The EMS providers in this study had not participated in the previous development of the e-triage tag. The 27 patients (figurants) were triaged, re-triaged, treated at the MCI scene, and transported to the hospital ED. All the information was captured and visualized for the EMS command unit, HICG, and RCC through the TriPoD web portal. Upon admission to the ED, seamless information sharing between the two devices (e-triage tag and hospital-e-tag) was managed by an instructor, a Registered Nurse employed at the ED. The real-time data were thereby continuously visualized through the TriPoD web portal at both the HICG and the RCC. A total of 137 participants took part in the exercise.

### Participants

Three participant groups were included in the study: (1) EMS providers; (2) the HICG, including physicians, nurses, and administrators at the hospital; and (3) the RCC, including designated duty officers, administrators, and disaster preparedness coordinators (Table 1).

**Table 1:** Descriptions of participants

	Profession
**EMS** ^1^ **providers ** ***n*** ** = 9**	RN^2^, Specialist
**HICG** ^3^ **-personnel ** ***n*** ** = 17**	Physicians (*n* = 5)
	RN (*n* = 4)
	Administrators (*n* = 5)
	Missing (*n* = 3)
**RCC** ^4^ **-personnel ** ***n*** ** = 11**	Physicians (*n* = 1)
	RN (*n* = 7)
	Administrators (*n* = 1)
	Missing (*n* = 2)

^1^EMS-Emergency Medical Services

^2^RN- Registered Nurse

^3^HICG-Hospital Incident Command Group

^4^RCC - Regional Command Center

### Data collection

Advanced approval for collecting data during the field exercise was obtained from both the EMS and hospital management. The specific goal of the data collection was to gain in-depth knowledge from the field exercise on the usability of TriPoD and to identify areas for further improvement in TriPoD design. Three observers collected data during the field exercise. The observers were stationed at the EMS command unit, the HICG, and the RCC. The observers were instructed to identify differences and/or similarities between TriPoD and the standard communication procedure. They were also instructed to listen to the participants’ reasoning regarding the functionality of using TriPoD. They were encouraged to document their reflections on the use of TriPoD in comparison with standard procedures. All observers had previous experience as EMS providers; two were PhD students in the field of EMS. Additionally, participants at the MCI site were encouraged to share their experiences with using the e-triage tag, scanner, and mobile application directly with the observer on the scene or by writing their reflections electronically or on paper. They were also encouraged to reflect on the system’s usefulness, utility, efficiency, and any logistical or technological challenges related to the TriPoD system. All data were collected through observers’ open-ended and free-form field notes and written comments from the participants. Discussions with the research team were conducted after the MCI scenario to clarify the written field notes. The first and last authors documented the discussions. The written descriptions from the participants in the field exercise were stored in a web-based document shared with the last author.

### Analysis

Before the analysis began, it was ensured that the collected data contained no personal information other than the participant’s profession. The written field notes by the observers, written reflections and notes by the participants in the field exercise, and text documented by the first and last authors were analysed thematically. The analysis was conducted according to the steps described by Kiger and Varpio [28]. Initially, the first and last authors read through the texts to gain familiarity with the collected material. The text was thereafter sorted into three groups: prehospital-, HICG-, and RCC-related text. The sorting was performed in Excel by categorizing the sentences from each group with different colors to achieve a structure. The three groups were analysed separately to explore and describe TriPoD usability from the three perspectives. The sorting of sentences in each group was followed by searching for and organizing them into preliminary themes. During sorting, the contents of prehospital, HICG, and RCC were similar; therefore, the groups were assembled. Nevertheless, the different colors for each group were retained to distinguish differences between them in the result presentation. The identified themes were subsequently refined and given appropriate labels. Finally, the analysis was completed by compiling and presenting the results by describing the three themes identified: *Real-time Information and Decision Support*,* Efficiency and Information Sharing*, and *Usability and Development Opportunities.* These three themes were then reviewed against the collected data to ensure consistency and accuracy. All steps were initiated by the first author, reviewed by the last author, and confirmed by the co-authors thereafter.

## Results

The identified themes were *Real-time Information and Decision Support*,* Efficiency and Information Sharing*, and *Usability and Development Opportunities.* These themes include perspectives on TriPoD from EMS, HICG and RCC personnel. Quotes underpinning the themes are displayed in Table 2.

### Real-time information and decision support

The participants in the field exercise acknowledge that real-time information plays a crucial role, as it provides instant updates on scene overview, patient status, triage categorization, and the location of patients. With this, the participants reasoned that TriPoD enabled proactive decision-making rather than only reactive responses. As TriPoD facilitated real-time tracking of patients, the participants at the HICG and RCC acknowledged that they were provided with information and the possibility of having a situation overview, which increased their knowledge of casualties, resource distribution, and patient flow to and within hospitals. The participants reasoned how traceability and immediate updates could reduce uncertainty, enabling efficient coordination between EMS providers, HICG, and the RCC. The ability to visualize dynamic changes ensured that each level of command had the necessary data to make informed, timely decisions. The observers’ field notes supported this reasoning among participants in the field exercise, stating that the real-time updates improved traceability and enhanced knowledge by displaying patient location, priority, and transport status through the TriPoD web portal. The TriPoD system provided a more accurate representation of patient distribution than standard communication. The observers noted that the TriPoD system’s web portal supported the commanders at HICG in preventing bottlenecks at the hospital, but this was not discussed among the participants.

### Efficiency and information sharing

The participants in the field exercise were reasoning around the importance of efficiency in triage and patient management, as it was fundamental to effective emergency response. They also discussed the importance of speed and accuracy in information sharing, which can have a direct impact on patient outcomes and overall healthcare system performance during an MCI. The participants perceived that the TriPoD system could significantly reduce the time required for information sharing from EMS providers to HICG and RCC. The observers confirmed this reasoning among the participants when they compared the information shared through the TriPoD web portal with radio communications from the field to the RCC and then to the HICG. However, the correctness of using TriPoD depended on whether EMS providers applied the e-triage tag and remembered to scan it when re-triaging and transporting the patient to the ED. Despite sometimes not having an accurate number of injured patients, the scanned patients received faster registration, a reduced administrative workload, especially at the HICG, and better preparedness at the receiving hospital. This was observed when the TriPoD web portal was not used, and administrators needed to make phone calls and send faxes between the ED and the HICG to obtain the number of patients arriving at the hospital. Additionally, the observers noted that using the TriPoD web portal at the RCC allowed for improved communication between different levels of command, ensuring that decisions were based on up-to-date and comprehensive data and information. Furthermore, the observers noted that using the TriPoD web portal at HICG could improve patient flow management and hospital capacity planning. However, these possibilities depend on whether seamless information sharing was correctly made from the TriPoD e-triage tag to the hospital e-tag device.

### Usability and development opportunities

Usability for users was discussed as a key factor among the participants, and the observers noted that a non-user-friendly e-triage tag used at the scene of the MCI resulted in the missed sharing of patient-related information with the RCC and HICG. The EMS providers sought a more intuitive and user-friendly e-triage tag to ensure they could quickly adapt and efficiently utilize it in high-stress environments, such as an MCI. The EMS providers’ feedback highlighted the importance of clear, simple, and efficient solutions, as well as the need for further development and adaptation of the TriPoDs e-triage tag. Suggestions for improvements included an alternative to using a mobile phone to scan the e-triage tag, as it was perceived as a hindrance to the workflow of retrieving the phone after applying the e-triage tag. The fastening of the e-triage tag was not secure, posing a risk of it falling off when moving patients. Once the e-triage tag was attached, it sometimes ended up on the underside of the arm, making scanning more difficult. Scanning must be easy, or it will not be performed according to the EMS providers. Scrolling quickly between triage colors when using the e-triage tag was helpful, but it must be ensured that the colors cannot be changed accidentally. Users were sometimes logged out of the mobile app, which is problematic since there is no time to log in between patients; the system needs to be more stable. Although these identified needs for improvement and development of the TriPoD e-triage tag used in the field exercise, the EMS providers concluded that the e-triage tag was easier to use than SMART Tag^®^ Cards [large colored cards displaying, for example the priority of an injured patient at an MCI]. They also acknowledged that TriPoD has strong potential and can be integrated into the broader EMS. At the HICG and RCC, they requested customization based on regional needs and different triage protocols, as well as better visualization of patient data, if possible. This would involve expanding the TriPoD system to include, for example, psychological triage and optimized patient tracking, such as tracking of surgical procedures within the hospital. HICG and RCC also wished for improved interface design for the web portal.

**Table 2:** Quotes underpinning the themes

Theme:	Real-time information and decision support	Participant
	I think it can make things easier and the work more transparent	RN specialist* (9)
	The future. Very good. Imagine being able to ‘see’ where the patients are on a large geographical incident site	RN specialist (1)
	Compared to the standard, they receive faster and more accurate information	Observer note (1)
	Discussion about how they can work more proactively rather than reactively when they have real-time information	Observer note (2)
	They seem to be more aware of the process between the incident site and arrival at the emergency department	Observer note (1)
	The real-time information made them aware of the expected arrival at various hospital locations	Observer note (2)
	Uncertainty when there are no updates on the web portal	Observer note (2)

Theme:	Efficiency and information sharing	Participant
	Can provide a clear overview of casualties in the incident area, offer quick real-time data and updates on the injured	RN specialist (4)
	Discussion about the importance of having accurate information	Observer note (1)
	More reliable information compared to the standard	Observer note (2)
	Possibility to plan patient flow at the hospital	Observer note (1)

Theme:	Usability and development opportunities	Participant
	It may have potential, but it wasn’t easy when nothing worked at the incident site, but then it worked	EMT** (7)
	Very good and positive. Easy for the arriving ambulance to scan the e-triage tag and access all the information. It has limitless development potential	RN specialist (1)
	It should be smooth and easy to use	EMT (5)
	The system MUST work without issues; otherwise, it won’t be used. Maybe mount the phone on the wrist to make it easier to use. Include vital signs in the app so that reevaluation can be done directly	EMT (10)
	Can’t something other than a mobile phone be used to scan the patients	RN specialist (5)
	Having to log in to the system is time-consuming and needs improvement	EMT (2)
	The web portal needs to be developed to become more intuitive	Observer note (2)
	Easier than the usual triage cards	RN*** (4)
	The triage tag tended to come off when patients were moved	Observer note (3)

* RN specialist Registered Nurse with specialist education

**EMT Emergency Medical Technicians

***RN Registered Nurse

## Discussion

In the event of an MCI, one of the most important tasks for healthcare management at different levels is to lead and allocate available resources to achieve the greatest possible patient benefit. To provide rapid and appropriate care, it is important to have competent disaster medical responders and leaders who can prioritize and sort to streamline efforts. The attributes of disaster medical leaders are crucial in ensuring an effective response and management during MCIs and disasters [11]. These attributes encompass a range of skills, characteristics, and qualities that allow them to lead and coordinate medical teams, respond to crises, and make critical decisions in high-pressure situations. It is emphasized that disaster medical leadership requires a set of competencies and a more nuanced, dynamic framework that can adapt to the unpredictable challenges of disasters [11]. Accurate, real-time visualized information is essential for making optimal decisions, regardless of the leadership’s effectiveness during an MCI or disaster. It is also important that the EMS organization collaborates with hospitals when using mobile technologies [29]. This study used mobile technology, and the findings indicated that the conditions for more effective decisions could be improved by using TriPoD e-triage tags and hospital e-tags rather than relying on phones, faxes, or paper documents. Through the TriPod Web portal, the ICs at the scene, HICG, and RCC received faster, concise, and continuous information about the number of injured people, their priority, and their locations. As discussed in a previous study, in an MCI, there is a need to identify those patients requiring life support and interventions from the scene of the injury and during transport to definitive care [30]. TriPoD has that possibility, but as this study revealed, the information’s correctness depended on EMS providers scanning the e-triage tag continuously; otherwise, the real-time information would not be accurate. At the hospital, HICG can track injured patients inside the hospital, and bottlenecks can be identified. For prevention, identifying possible bottlenecks could provide opportunities for HICG and RCC to better plan for patient transportation between different hospitals and within various hospital units.

A key task for ICs at different levels at an MCI is to gain an overview, and there is a need for more effective communication between EMS providers and commanders at different levels and locations [31]. At the same time, EMS providers are responsible for performing triage to prioritize the injured based on the severity of their conditions. The TriPoD system has been developed to work regardless of which triage algorithms are used. In the TriPoD system, the e-triage tag serves as the carrier of information, meaning it collects and shares data in real-time. When the injured person arrives at the hospital’s ED, the information from the e-triage tag can be shared seamlessly with hospitals that use e-tags to track their patients. In this study, information was shared from the e-triage tag at the ED when the EMS providers handed over the patient, and thereby, the HICG received instant information through the web portal. This seamless information sharing can support the IC’s decision-making. During the evaluation, personnel at the HICG suggested that the e-triage tag could provide additional information that would be helpful to them. There are no direct limitations on the amount of information that can be stored in the e-triage tag. But, the question is how much information EMS providers can expect to retrieve, note, and share while operating in a high-stress environment such as an MCI. Regardless, thoughts and wishes will be considered in collaboration with end-users when further developing the TriPoD system, as they are the ones who will use it.

In high-stress, time-sensitive situations, leaders must rely on real-time information, which is often incomplete, and make decisions that immediately affect the response. Decision support systems must assist leaders by providing and visualizing structured information in real time, which helps them evaluate the impact of different choices and decisions [13, 32]. Based on the study’s findings, it is reasonable to assume that TriPoD could serve as a system for information sharing between different levels of management. However, according to the end-user discussions and evaluation in this study, further development of the e-triage tag and the web portal is needed. The design and development of TriPoD has used an AR design [24], which is suitable for developing and modifying practices in healthcare settings. AR is cyclical and involves development, evaluation, redevelopment, and replanning [33]. Therefore, based on the findings of this study, the dialogue between the research team, information technology producers, and end-users will continue.

Regardless of the need for further development of the TriPoD system, the e-triage tag and the web portal findings from the MCI field exercise demonstrated how the TriPoD system facilitated communication and information sharing across different management levels. This contributed to improved real-time coordination during the field exercise of the MCI. Since information sharing, communication, and coordination are critical in MCI management, disaster medical leadership requires continuous updates and refinements of knowledge and skills as new technologies, methodologies, and challenges emerge. TriPoD could be a valuable tool in healthcare management at different levels during an MCI or disaster. Nevertheless, an enhanced competency framework for command leadership should encompass leadership, communication, and situational awareness and integrate decision support and the technical and medical skills essential in disaster settings [13].

## Limitations

This study has a qualitative design and is a part of an AR study plan [24]. AR research employs a cyclical study design, involving development, evaluation, redevelopment, and replanning [33]. While AR promotes the transferability of findings, the generalizability of results remains context dependent, as is the case with other qualitative research. The TriPoD framework acknowledges variations in EMS structures and resources across local, regional, and national settings. Its core components, triage, emergency care, and transport to appropriate facilities, are fundamental to global prehospital emergency care. Therefore, it is reasonable to assume that TriPoD could be used in different EMS contexts. Using non-structured observations for data collection may introduce bias, as the observer’s beliefs, expectations, or prior experiences may influence how they record their field notes [34]. Structured observation protocols could have provided clearer insights into the frequency of various observations; however, this was not the primary aim of the study. Furthermore, the use of validated questionnaires could have provided additional data, but we prioritized collecting participants’ immediate reflections. This decision was based on concerns about potential non-response if surveys were distributed after the exercise. However, this approach limited the opportunity to capture more structured and comparable data. Another limitation of using observations for data collection is that observers can capture only what is visible or audible [34], meaning they may miss important contextual or internal factors that influence the participants’ decision-making processes. However, the observers in this study all had contextual knowledge as EMS providers and researchers, meaning they could understand participants’ behaviors, interactions, and decision-making processes. Despite the limitations of this study, the knowledge generated can be applied to the development of TriPoD, and it also addresses the complexity of management at various levels of an MCI, as well as the importance of seamless, real-time information in decision-making. Furthermore, the study contributes knowledge on using mobile applications and web technology to support real-time information and triage during an MCI.

## Conclusion

The study demonstrated that seamless information sharing from the scene of an MCI enhances reliable communication and management efforts. Although TriPoD shows strong potential for improving MCI response and management, further development, evaluation, and research collaboration with intended users are essential. Collaboration with end-users ensures that the research outcomes are not only theoretically grounded but also feasible, sustainable, and can be impactful in improving practice.

## Electronic supplementary material

Below is the link to the electronic supplementary material.

Supplementary Material 1

## Data Availability

The datasets used and analyzed during the current study are available from the corresponding author upon reasonable request.

## Abbreviations

AR: Action Research
ED: Emergency Department
EMS: Emergency medical services
HICG: Hospital Incident Command Group
ICS: Incident Command System
MCI: Mass casualty incident
RCC: Regional Command Center
TriPoD: Triage, Position, and Documentation
